# Does Disease-Irrelevant Intrathecal Synthesis in Multiple Sclerosis Make Sense in the Light of Tertiary Lymphoid Organs?

**DOI:** 10.3389/fneur.2014.00027

**Published:** 2014-03-11

**Authors:** Mickael Bonnan

**Affiliations:** ^1^Service de Neurologie, Centre Hospitalier F. Mitterrand, Pau, France

**Keywords:** multiple sclerosis, lymphoid tissue, antibody response, cerebrospinal fluid, Epstein–Barr virus

## Abstract

Although partly disease-irrelevant, intrathecal immunoglobulins (Ig) synthesis is a typical feature of multiple sclerosis (MS) and is driven by the tertiary lymphoid organs (TLO). A long-known hallmark of this non-specific intrathecal synthesis is the MRZ pattern, an intrathecal synthesis of Ig against measles, rubella, and zoster viruses. This non-specific intrathecal synthesis could also be directed against a wide range of pathogens. However, it is highly problematic since brain TLO should not be able to drive the clonal expansion of lymphocytes against alien antigens that are thought to be absent in MS brain. We propose to explain the paradox of non-specific intrathecal synthesis by discussing the natural properties of TLO. In fact, besides local antigen-driven clonal expansion, circulating plasmablasts and plasma cells (PC) are non-specifically recruited from blood and gain access to survival niches in the inflammatory CNS. This mechanism, which has been described in other inflammatory disorders, takes place in the TLO. As a consequence, PCs recruited in brain mirror the individual’s history of immunization and intrathecal synthesis of IgG in MS may target a broad range of common infectious agents, a hypothesis in line with epidemiological data. Moreover, the immunization schedule and its timing may interfere with PC recruitment. If this hypothesis is correct, the reaction against EBV appears paradoxical: although early infection of MS patients is systematic, intrathecal synthesis is far lower than expected, suggesting a crucial interaction between MS onset and timing of EBV infection. A growing body of evidence suggests that the non-specific intrathecal synthesis observed in MS is also common in many chronic CNS inflammatory disorders. Assuming that cortical TLO in MS are associated with typical sub-pial lesions, we have coined the concept of “TLO-pathy” to describe these lesions and take examples of them from non-MS disorders. Lastly, we propose that intrathecal synthesis could be considered a strong hallmark of CNS TLO and might be used to monitor future TLO-targeted therapies.

## Introduction

The intrathecal synthesis of immunoglobulins (Ig) and oligoclonal bands (OCB) is an early occurring event in the course of multiple sclerosis (MS), and once acquired, persists essentially unchanged throughout life, whatever therapies are undertaken [see review in Ref. (1)]. As a consequence, intrathecal Ig secretion is still a major supportive diagnostic argument. However, the antigens targeted by these intrathecal Ig are often considered to be largely unknown and studies have yielded contradictory results (2). The lack of specificity against the three major myelin proteins does not preclude any other specificity (3), either against other minor myelin proteins or brain antigens, or against foreign antigens (viral antigens for example). Indeed, the failure to find a major antigen for an intrathecally synthesized Ig may not relate to a nonsense antibody production but instead may reflect the molecular complexity of the CNS and the presumed antigenic target. Studies without any *a priori*, which are better suited to revealing unknown specificities, have confirmed the existence of an immense pool of targets against other brain membrane proteins, lipids, and glycolipids [see Ref. (3)]. Paradoxically and despite these huge efforts, the puzzling problem of non-brain targets directed against a virus in the absence of brain infection seems irreconcilably contradictory with an intrathecal antigen–antibody selection. Although the concept of molecular mimicry has long been debated [review in Ref. (4)], epidemiological data suggest that mimicry alone cannot explain the intrathecal synthesis that occurs against so many infectious agents [(5, 6), see below]. Here, we reassess the pathophysiological consequences of this non-specific (disease-irrelevant) intrathecal reaction in MS in the light of the most recent immunological data and develop a new concept with great explanatory potential by bringing together the apparently contradictory data in the literature.

First, we comprehensively review the non-specific intrathecal reaction in MS. While the classical “MRZ pattern” is the hallmark of this non-specific reaction, we argue that MRZ is simply part of a broader non-specific reaction involving many non-brain targets, such as most of the common infectious agents.

We then explore the conditions necessary to produce and maintain an intrathecal reaction. We first use the example of peripheral (non-brain) lymphoid organ physiology to demonstrate how a sustained antibody secretion may take place in a given lymphoid organ, even if the mature B-cells originate from a different immune compartment. We posit that disease-irrelevant Ig secretion is a common feature of tertiary lymphoid organs (TLO) in various inflammatory disorders. We then link these observations to the fact that intrathecal synthesis needs the presence of cortical TLO, implying in turn a non-specific intrathecal synthesis.

We then explain why virtually all the CNS disorders associated with intrathecal synthesis may also harbor some non-specific synthesis, and provide evidence for this reaction and its extent in multiple CNS inflammatory disorders. Since intrathecal synthesis involves CNS TLO and bearing in mind that TLO in progressive MS are associated with sub-pial lesions (7), we propose the pathological concept of “TLO-pathy” and provide examples from chronic CNS infections.

Finally, we return to the issue of MS. Since non-specific intrathecal synthesis is directed against many infectious agents, the question arises as to whether an immunization schedule could be used to accurately assess the time of MS onset? Secondly, although EBV infection precedes MS in virtually all patients, the intrathecal reaction against EBV is paradoxically very low. This might either illustrate the role of the immunization schedule or a specific relationship between EBV and MS.

In practice, intrathecal synthesis in MS informs the clinician mostly of the persistence of CNS TLO at the pathological level. Such information obtained from a simple lumbar puncture could help in the monitoring of future treatments targeting CNS TLO.

## The Concept of Non-Specific Intrathecal Synthesis in MS

### The “MRZ” pattern – a typical non-specific intrathecal response

Barring a still unknown cross-reactivity from viral particles and the brain, the “MRZ” reaction, which is the intrathecal synthesis of Ig against measles, rubella, and varicella-zoster virus, has long been considered to be highly specific of MS, but does not target the brain (8). The IgG fraction that belongs to this specific response in the CSF is estimated to represent <2% of the total amount of CSF IgG (8–10) and only a minor fraction of total OCB (11–13), but it is one that has a major diagnostic specificity. Moreover, the MRZ reaction is observed in MS patients although none have been reported to have intrathecal production of these viruses or to suffer from encephalitis involving any of these viruses. The proportion of MS patients having an elevated antibody index (AI) against ≥1 of these viruses is 89% (8), and increases over time and MS evolution. About one-third of patients also react against Herpes virus (MRZH). The MRZ reaction, which is sometimes present in the absence of OCB, is more frequent in clinically isolated syndromes (CIS) who will soon convert (14) and its proportion increases in OCB-negative patients during follow-up (15). In a retrospective study of MRZH in OCB-negative patients, 4 (18%) of 22 patients having been diagnosed with MS within the last 5 years had at least one elevated AI, compared to 17/28 (61%) in those with a longer disease duration (15).

Intrathecal synthesis is poly specific in about a quarter of MS patients: one elevated AI (against M, R, Z, or H) in 22%, two AI in 17%, three AI in 4%, and four AI in 2% (15). The number of elevated AI strongly correlated both with age at spinal tap and disease duration (15). Immunosuppressive treatments (mitoxantrone and azathioprine) seem ineffective to prevent persistence of the MRZ pattern (16). Titers of AI against MRZ correlated with the T2 load in MRI, significantly for measles AI and with a trend for rubella and zoster AI (17). However, this correlation is limited to the early phase of the disease since intrathecal Ig secretion is rather stable over time, although white matter T2 load increases (17), but no data is available to correlate with gray matter lesions. Unlike in controls, no decline in AI levels occurs with age in MS patients either considering serum or CSF titers, and there is even a slight increase (18, 19). Moreover, there is a trend to a higher proportion of elevated AI in SP-MS than in RR-MS patients (20).

### Broadening the MRZ pattern to multiple infections

Beyond the MRZ pattern, high intrathecal synthesis against many other infectious agents has been reported in MS: rotavirus (21); toxoplasmosis in 10% (8, 10); herpes in one-third of cases (8, 21); *Chlamydia pneumonia* in 20–82% (9, 11, 20, 22–25); HHV6 in 20–30% (26, 27); *Borrelia burgdorferi* in 26% (28); mumps (29); influenza B (29); rotavirus (29); adenovirus (30); and vaccinia (30, 31). In each study, seroprevalence for infection was the same in both MS and controls, whereas controls had no specific intrathecal reaction. Unfortunately, some of these older studies used heterogeneous methodologies and some of them should be validated with recent stringent criteria (10). The same gradient of frequency from RR-MS to SP-MS is observed with intrathecal productions against many infectious agents (20, 22). This polyspecific intrathecal IgG response in MS mirrors the individual’s history of previous infections and immunization and depends on the local prevalence of each disorder. For example, a lower proportion of rubella-AI is observed in MS patients from Cuba than those from Germany, in line with a lower incidence of rubella infection (6). Interestingly, the sex-ratio is far lower (M:F = 1:6) than expected (M:F = 1:1.9) in Cuban patients synthesizing intrathecal rubella antibodies, a finding reliably explained by immunization campaigns directed toward females (but not males) in a context where natural infection is very low (6). In Czech patients, a high proportion of AI against *B. burgdorferi* has been observed (up to 26%) (28). In fact, the rate of intrathecal reaction against a given germ correlates with the rate of seroprevalence, very highly seroprevalent infections (about 90% for measles and rubella) having the higher rate of intrathecal reactivity whereas low prevalence of HSV is associated with scarce reaction (29). This former epidemiological subjection and the broad range of infectious agents potentially involved plead against a trivial cross-reactivity of intrathecal synthesis with brain antigens. We hypothesize that a high level of intrathecal reaction against *all* the common antigens (infectious/vaccinal or not) is probably common in MS and could throw light on the pathophysiology of MS.

## Key Properties of CNS Tertiary Lymphoid Organs Explain Intrathecal Synthesis, Including Non-Specific Secretion

### Plasma cells may locate not only in bone marrow but also widely throughout the peripheral lymphoid organs

The protracted presence of intrathecally secreted IgG against infectious agents sparing brain supposes the non-specific recruitment and survival in the intrathecal compartment of long-lived plasmocytes originating from a different immune compartment, where the B-cell response against infectious agents previously took place. The ability to develop an immune response in a compartment and to transfer specific B-cells to another unexposed compartment is a common immunological feature outside of the brain (32, 33).

**Figure 1:**
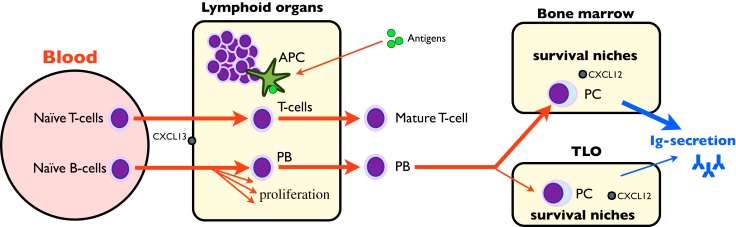
**Schematic Ig synthesis pathway**. Naïve B-cells are classically recruited in lymphoid organs where they undergo somatic hypermutation, proliferate, and recirculate as short-lived plasmablasts (PB). PB migrate to bone marrow, differentiate to long-lived plasma cells (PC), and synthesize the bulk of plasmatic IgG. However, secondary/tertiary lymphoid organs may also retain PC in survival niches, where they participate in IgG synthesis.

During immune activation in the periphery, naïve B-lymphocytes encountering antigens are committed to plasmablasts and undergo hypermutation in germinal centers of secondary lymphoid organs. Then, specific plasmablasts appear in the blood for a few days on their way to the survival niches, where they differentiate into plasma cells (PC) (32) (Figure 1). Most of the niches are situated in bone marrow (a primary lymphoid organ) (34). Since the frequency of PC in bone marrow is constant throughout life (about 0.5%), newly formed plasmablasts migrating to bone marrow compete with PC already occupying survival niches (34). Thus a majority of newly formed plasmablasts arriving in the bone marrow fail to locate to an appropriate niche (32). This competition for a limited number of survival niches may play a key role in the regulation of serum antibody levels and has also been demonstrated in different animal species (34). However, niches are also available in peripheral secondary and TLO where they display the ability to retain the newly formed PC (32, 35–39). The retention frequency of PC for a given specificity strongly depends on the lymphoid organ: for example, PC against tetanus toxin preferentially reside in secondary lymphoid organs such as tonsils, and their antibody titers stem from this compartment (35). It also depends on the route of immunization, making specific B-cells more numerous in the draining lymph nodes although they are present to a lesser extent throughout the lymphoid system: for example, a massive rectal immune response is obtained after rectal immunization, yet a rectal response, although minor, is also obtained after gastric or systemic immunization (33). The frequency of disease-irrelevant PC in TLO does not depend on the underlying disorder: for example, the same anti-tetanus toxin IgG concentration is obtained in the culture of synovial extracts (containing TLO) from osteoarthritis or rheumatoid arthritis (38) as in chronic graft rejection (37). It thus appears that non-specific Ig secretion is a natural condition of peripheral TLO.

### Are antibody-secreting cells hosted in TLO survival niches, thus explaining the MRZ pattern by non-specific niches offered by CNS inflammatory lesions?

**Figure 2:**
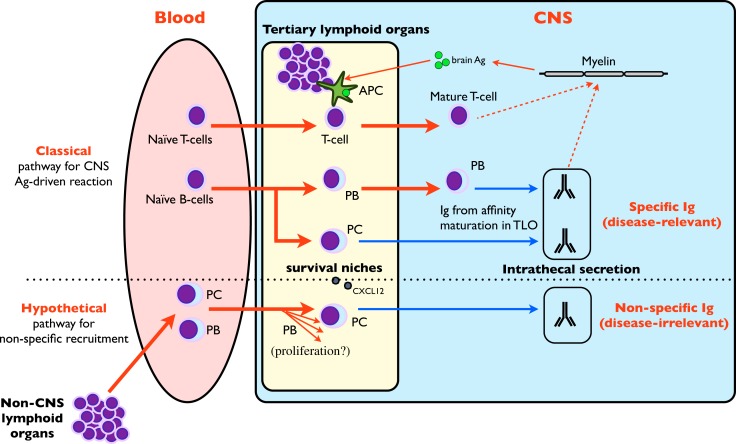
**Role of tertiary lymphoid organs in intrathecal synthesis**. CNS TLO are able to mount a classical antigen-driven T and B-cell clonal response that is responsible for intrathecal synthesis against brain-borne (and possibly disease-relevant) antigens (1, 46–48) (upper part of figure). A hypothetical pathway (lower part of figure) may involve the non-specific recruitment of circulating plasmablasts (PB) and plasma cells (PC) from blood by CNS TLO. These disease-irrelevant PB may further differentiate after proliferation to PC, home in on survival niches provided by TLO and maintain the non-specific intrathecal Ig synthesis.

Depending on the primary antigenic stimulation, the estimated half-life of long-lived PC varies from 11 years (tetanus) to more than 100 years (measles, mumps), but long-lived PC expelled from niches during competitive process undergo apoptosis (39). In fact, prolonged survival necessitates an anti-apoptotic environment provided in the survival niches by multiple factors (multicomponent PC niche) [review in Ref. (32, 39)]. Although the complex factors conditioning recruitment, differentiation and survival of PC in niches are still incompletely deciphered and vary depending on the different immune compartments (32), CXCL12 expression by stromal cells and vasculature is the major determinant to retain blood plasmablasts in bone marrow (32, 34). In MS, CXCL12 is elevated in CSF, secreted by astrocytes (40), and expressed in the endothelial lumen of lesions in the vicinity of lymphoid infiltrates (41) where PC infiltrates are usual, suggesting that MS brain is ideally equipped to retain non-specific circulating plasmablasts. Although no data is available for meningeal lesions, one could also expect a meningeal CXCL12 expression similar to that in deep lesions (41). The presence of PC inside white matter lesions and meninges in MS has already been described (42, 43). According to the previous hypothesis, peripherally activated plasmablasts specific to infectious agents may egress from blood owing to CSF attractant chemokines secreted in brain lesions (i.e., CXCL12), reach survival niches in the CSF and brain, differentiate to PC without any antigen (re)challenging, and finally secrete non-specific intrathecal IgG over a long period (Figure 2). Another mode of interaction of PC in survival niches may involve T-cells, since PC depletion in lymphoid organs following alemtuzumab treatment may be associated with the loss of other cells supporting PC survival (36). Although these data were obtained in experimental settings unrelated to MS, they offer an attractive explanation of the (slight) repression of intrathecal synthesis observed for the first time in MS patients treated with natalizumab (44, 45).

Similar to MS, the extreme versatility of disease-relevant antibodies and the presence of disease-irrelevant antibodies like anti-tetanus toxin have been directly associated with TLO function [chronic graft rejection (37), rheumatoid arthritis (38)]. We suggest that the intrathecal production of disease-irrelevant antibodies may be due to antibody-secreting cells harvested in survival niches of TLO, as in numerous other peripheral disorders (37, 38). Definitive proof – but hard to obtain – would be obtained by using *in vitro* cultures of explanted MS TLO and assaying disease-irrelevant antibodies in the supernatant. Moreover, since most experiments to date concerning Ig secretion in body compartments have been done with anti-tetanus toxin, it would be interesting to evaluate AI anti-tetanus toxin in MS patients, in whom high levels could be expected to occur frequently.

Under these assumptions, a protracted polyspecific Ig response would indicate an enhanced pre-existing B-cell promoting environment. The MRZH pattern mainly mirrors the commonest infections, i.e., those that are the most likely to select abundant specific plasmocytes, which are subsequently recruited non-specifically by the brain TLO. Moreover, intrathecal antibody-secreting cells recruitment should be progressive and achievement of a full MRZ pattern may take years or even decades. This is in fact observed in clinical studies where mean age at spinal tap and disease duration correlate with the number of elevated antibody indices (15). Half of the patients having fewer than 5 years of disease duration have no elevated AI (MRZ), whereas 70% of those with more than 10 years duration have AI ≥2 (15). It would be of particular theoretical interest to confirm the MRZ pattern in MS patient populations where OCB prevalence is lower, in association with HLA DRB1*04 (15).

The presence of a polyspecific intrathecal synthesis as soon as the index event occurs suggests that an enhanced B-cell-promoting environment exists before or at the time of the first event, and gives a high probability of a chronic inflammatory process already underway at the moment of the first clinical symptom (6). This raises two further issues: (1) a polyspecific intrathecal response might also be associated in non-MS patients susceptible to CNS TLO (e.g., in chronic CNS infections); (2) the schedule of peripheral non-specific infections (e.g., MRZH infections) might interfere with the ancientness of the TLO-leading disease (i.e., MS or brain infection) in the risk of developing a bystander non-specific intrathecal synthesis.

## Non-Specific Synthesis is also a Feature of Non-MS CNS Disorders Harboring Intrathecal Synthesis

### Non-specific vs. infection-related intrathecal antibody synthesis

The fraction of a specific antibody response against an infectious agent within the complete intrathecal IgG response is called the Specific Fraction (*F*_s_) (49). For example, the calculation of an *F*_s_ value for measles at 2% means that 2% of the total intrathecal IgG response is directed against measles. Neuro-infections are expected to be associated with very high *F*_s_ against the virus: *F*_s_ are 8.8% (3.5–12.5%) for HSV in HSV encephalitis (HSVE), 18.8% (11.8–27.5%) for measles in subacute sclerosis panencephalitis (SSPE) (49, 50) and 45% (13–73%) for VZV encephalitis (51). Unfortunately *F*_s_ values for relevant infectious agents are not available in other infectious settings such as neuro-syphilis and neuro-borreliosis. In EBV-associated post-transplantation brain lymphoma, median *F*_s_ anti-EBV is 27.8% (14–53.6%) (52).

**Figure 3:**
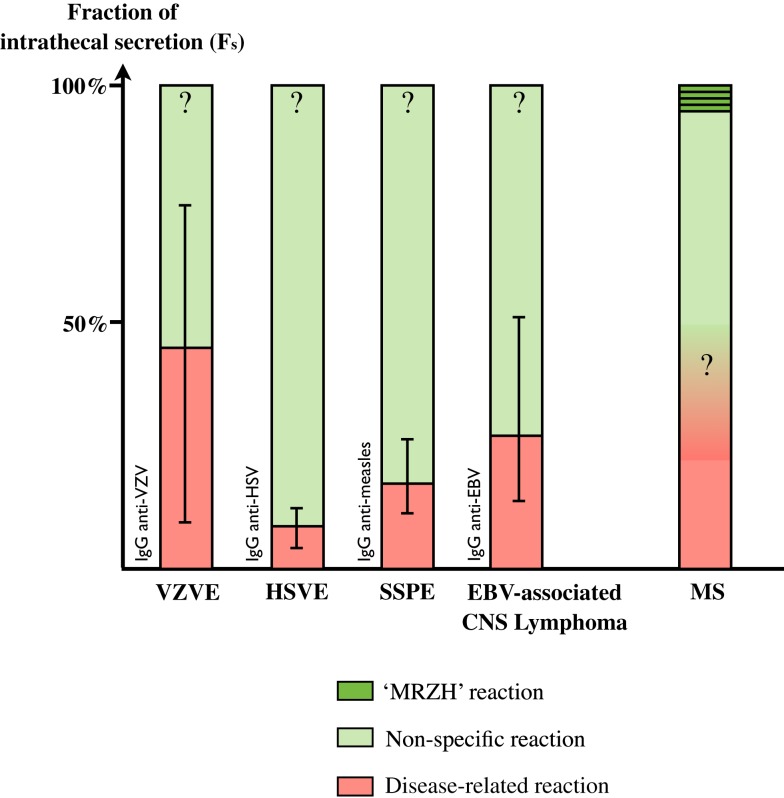
**Fraction of intrathecal Ig secretion in various disorders**. Disease-related Ig secretion is minor even in infectious disorders (See Ref. in text). Intrathecal synthesis is mostly dominated by non-specific synthesis, including an MRZH pattern, which represents a minor proportion. Disease-related secretion in MS and MRZH patterns in infections are as yet unknown.

In MS, each specificity in the MRZH reaction typically retains a very low median *F*_s_ of 0.2–1.3% (ranging from 0.03 to 5.3) (49, 51, 52) and comparable results are obtained for *F*_s_ anti-EBV (52). These specific *F*_s_ results in neuro-infections are about 40-fold higher than those found in MS patients without overlap. Interestingly, in two cases of HSVE, median *F*_s_ anti-VZV were 0.3–4.9%, in the same range as those of MS patients (51). In other words, about 80% of the intrathecal Ig in infectious pathologies are directed against non-causative antigens (10, 49, 50), i.e., a non-specific intrathecal synthesis is very common even in neuro-infections (Figure 3).

The specific antibody fraction of the total IgG in blood (*R*_s_) corresponds to the intrathecal fraction of antibodies in CSF (*F*_s_) (49). For example an *R*_s_ value for IgG against measles of 0.1% means that a 0.1% of the total IgG pool is directed against measles. Median *R*_s_ values range from 0.06 to 0.19% (0.01–1.4%), which is a very low fraction of total blood IgG and does not differ between MS patients and controls (8). The median ratio *F*_s_:*R*_s_ for MRZ is 3–3.5 with a wide range extending from 0 to 47.4 (10, 49), confirming a more intense intrathecal synthesis of specific antibodies.

The variability in the amount of specific intrathecal synthesis between neurological infections and MS patients as compared to blood deserves discussion. The amount of intrathecally secreted specific antibodies should be proportional to the number of intrathecal PC. If we consider that circulating PC are non-specifically and randomly selected from blood to home to CNS TLO, the relative proportion of each specific IgG synthesis in CSF should grossly parallel their proportion in blood. For example, if IgG secretion in blood against viruses V1 and V2 is 0.5 and 1% of the total blood IgG, one could expect the same proportion and the same ranking of IgG secretion against those viruses in CSF. Yet this is not the case. The ranking of specific antibodies concentrations in blood and CSF differs in 67% of MS patients, confirming that CSF IgG secretion does not simply mirror blood secretion. Intrathecal synthesis of the specific antibodies occurs independently from each other (49). We propose two non-mutually exclusive explanations. A first hypothesis involves the differential intrathecal proliferation of specific B-cells after being recruited from blood but before being committed to terminal differentiation into PC, owing to a favorable intrathecal lymphoid environment. Unfortunately, no data is available about the clonality of the IgG involved in the MRZ response. A second hypothesis posits a non-random brain homing of circulating plasmablasts. It seems unlikely that plasmablast homing is driven by IgG specificity. Rather, the critical intensity of plasmablast/PC recruitment to brain owing to the intensity of the peripheral immune response to infection could be involved. For example, since immunity is now largely obtained by vaccination, which has been used for almost 30 years, rather than by infection; has intrathecal synthesis against measles changed over time? A study suggests that patients with a history of measles infection are more prone to have detectable anti-measles antibodies in the CSF (89 vs. 67%) and at higher levels than those who have been vaccinated (19). Unfortunately, rates of CSF anti-measles secretion were not correlated to age at infection and an exact determination of intrathecal synthesis was lacking, so further studies on this theme are required.

### Polyspecific intrathecal synthesis in non-MS disorders

The polyspecificity of the intrathecal response is not restricted to MS but is also common in response to infections [see above (49)] or non-MS CNS inflammation.

(a) **Autoimmune disorders**. In large control groups of non-autoimmune and autoimmune non-MS neurological disorders, a monospecific MRZH response was present in respectively 3/37 (8%) and 3/16 (19%) while multiple responses were never observed (15, 25, 28). In one series, 1/1 Sjögren and 1/1 Wegener and 3/9 lupus had an MRZH response (including two with intrathecal synthesis of anti-DNA) (53). In a series of 17 primary Sjögren’s syndrome, 11 had OCB, 6 had a high IgG index, 13 had a high IgM index, 8 had at least a reaction (immunoblot) against one specificity of the MRZH pattern, but none had anti-MBP (54). Interestingly, many series harbor a frequency of abnormal specific AI at low frequency in non-MS disorders but at intermediate frequency in the autoimmune non-MS disorders group (22, 28). When focusing upon paraneoplastic neurological syndromes (characterized by anti-Hu, -Yo, -Ri, -Ma, -Ta or -Tr antibodies either in serum or CSF, and by a CNS acute inflammation), a monospecific MRZ response was present in 7/34 vs. 0/42 in MS, whereas a bispecific or a trispecific response was obtained in none (0/34) of these syndromes vs. 37/42 MS patients (55). A bispecific response was obtained in 1/20 neuromyelitisoptica (NMO) (16). No intrathecal synthesis was observed against CMV or EBV in a large group of amyotrophic lateral sclerosis (ALS) and chronic inflammatory demyelinating polyneuropathy (CIDP), a setting in which intrathecal synthesis is usually absent (56). In CNS lymphoma, AI is commonly elevated for EBV but not against measles, yet results obtained with EBV may obscure the interpretation due to its dedicated role in lymphoma’s pathophysiology (52).
(b) **Infectious disorders**. In a few cases of neuro-borreliosis, neuro-syphilis, or neurotuberculosis, a MRZ synthesis occurs against one species in a few percent and below 0.1% for M + R + Z (8, 57, 58). However, neuro-syphilis should be studied differently. There has been one case of neuro-syphilis showing elevated AI for MRZ and mumps (59). In HIV infection, apart from elevated AI against HIV itself and HSV, *C. pneumoniae*-specific OCB are found in 3/5 patients, HSV OCB in half of all patients, as well as OCB against CMV and toxoplasma (25, 58, 60). In a series of 17 HTLV-1, only one showed a complete MRZ response (57), but none did in a series of 11 HSVE (61). In another series of nine HSVE, all had a persistent response against VZV and one had an intrathecal synthesis against measles (62). In the latter patient, synthesis against measles was absent at week 1, appeared at week 4, and persisted at year 4.5 (62). In a series of seven children having suffered from HSVE when they were younger, 3/5 who underwent lumbar puncture more than 8 years later had a partial or complete MRZ pattern but none did of those who underwent lumbar puncture in the same year (63). Moreover, IgG index increased over time, reaching a maximum at 1–2 months, then mostly remained elevated years later (63, 64). Systematic studies are lacking but in a single case of HSVE, an initially high AI for herpes subsequently abated whereas AI against multiple viruses (measles, parainfluenza, influenza, and adeno) increased (63). A systematic study of 10 mumps meningitis revealed a combination of multiple intrathecal reactivity against measles, herpes, adenovirus, or rubella in most patients, in the same range as that observed in MS patients (65). In one case, non-specific reactivity against measles and herpes was already present at day 1 whereas mumps reactivity appeared at day 10 (65). In acute neuro-borreliosis, the presence of an MRZ pattern is documented in up to 7% of patients, whereas in late neuro-borreliosis, a considerable number of OCB were not attributable to *B. burgdorferi* (66), and an MRZH pattern with three specificities was observed (58).
(c) **Genetic disorders**. The systematic study of CNS genetic disorders may open up an unexpected window of reciprocal intrathecal autoimmunity. A seminal work in Batten’s disease, which is a purely degenerative CNS genetic disorder, demonstrated a high level of intrathecal antibodies against GAD (glutamic acid decarboxylase) in patients and in the murine model (67). Results are even more interesting in X-linked adreno-leukodystrophy (ALD). OCB was found in only one patient in 13 with typical ALD but was absent in asymptomatic patients (68). However intrathecal synthesis was present in all but 1 of the 14 ALD patients and in none of the 12 asymptomatic patients (68). This synthesis predominates in IgA in all cases but one, but only rarely in IgG or IgM. Interestingly, intrathecal synthesis was found negative in CSF sampled before the onset of brain symptoms and later became positive. The MRZ pattern was absent in all cases, but none had a follow-up exceeding 2 years after onset of brain symptoms (68). Moreover, the inflammatory component in ALD is a secondary event, mostly localized in the center of lesions but sparing the edges, contrary to MS (68). The two latter examples suggest that whatever the chronic brain lesion, an intrathecal non-specific inflammation may be triggered.

In conclusion, there is a lack of large systematic studies of MRZH (and other specificities), AI in *chronic protracted autoimmune brain disorders* and especially in *chronic brain infections*, taking into account age at infection onset, immunizations prior to and during the infections and duration of disease. However, the above-mentioned data strongly suggest that a polyspecific intrathecal synthesis occurs more commonly than thought in association with *long-standing* infection/post-infection intrathecal specific response. In line with the progressive enrichment of the MRZ pattern over time in MS, these observations support our main hypothesis postulating a (slowly) progressive recruitment of non-specific PC over time.

### Could we use chronic neurological infections as a model for the study of non-specific intrathecal immune synthesis?

The above-mentioned arguments support the existence of a non-specific intrathecal reaction associated with neurological infections. Moreover, this non-specific reaction takes years to develop, since it is more frequent in chronic infections and in older MS patients. The factor triggering MS and the reason why intrathecal inflammation fails to clear in MS are still unknown. From a theoretical point of view, the study of these chronic neurological infections may open up a major opportunity that is unavailable in MS investigations by controlling two key points: infection triggers a specific (and non-specific) intrathecal inflammation and antibiotics cure the cause. This may allow the study of the natural clearance of CNS inflammation after the end of the cause, which may provide information about the late progressive stages of MS.

Nevertheless, from a technical point of view, it now seems very difficult to document non-specific intrathecal synthesis in chronic neurological infections. Thanks to medicine and hygiene, most of the above-mentioned chronic infections (SSPE, HTLV-1, neuro-syphilis, chronic neuro-borreliosis) have now practically disappeared from westernized countries. Other chronic protracted CNS infections such as HTLV-1, neurocysticercosis, and trypanosoma, which are associated with a strong specific intrathecal synthesis, are all neglected diseases from tropical areas, and medical research into them and their treatments have been sidelined. HIV infection would lend itself to the long-term study of the progressive enrichment of intrathecal synthesis, but immune dysfunction might interfere during follow-up.

### Rethinking sub-pial lesions of progressive MS in the light of non-specific intrathecal inflammation: Introducing the concept of “TLO-pathy”

**Figure 4:**
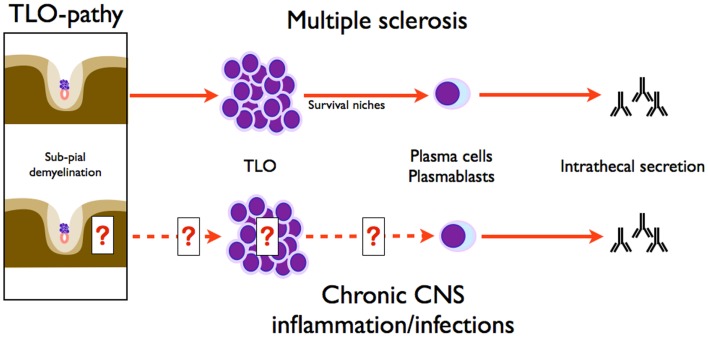
**Symmetry of intrathecal synthesis paradigms in MS and chronic CNS inflammation/infections**. Intrathecal synthesis in CNS infection implies plasma cells, and as demonstrated herein, needs survival niches supported by TLO. Sub-pial demyelination is associated with TLO, as classically known in MS, but also in chronic CNS inflammation and infections. Therefore, sub-pial demyelination induced by TLO is not a hallmark of MS and defines the concept of TLO-pathy.

Studying intrathecal synthesis in non-MS disorders could be far from futile. Although proof of CNS TLO is lacking in many disorders associated with intrathecal synthesis, owing more to the recentness of the discovery of TLO than to negative studies, no alternative theory better explains the long-lasting intrathecal synthesis observed years after neuro-infection healing, for example after neuro-syphilis, borreliosis, or HSV (Figure 4) (62, 66, 69). Do cortical TLO exist in chronic infections and are they associated with congruent sub-pial lesions as in progressive MS? In a series of nine patients suffering from different types of neuro-infections, two were proved to have meningeal B-cell aggregates (one tuberculous meningoencephalitis, one syphilitic meningitis) (7). Furthermore, sub-pial demyelination (layer I) was observed in syphilitic meningitis (7). In the event of independent study replication, sub-pial lesions associated with cortical TLO should no longer be considered as typical of progressive MS, but should rather be seen (at least in some cases) as bystander lesions induced by TLO independently of the causal disorder: infection, inflammations, or MS (1). Pathophysiology may involve by-products of TLO function like cytokines (TNFα, IFNγ, etc.), which were shown in vitro to have a noxious effect on cultured brain cells owing to antibody-independent toxicity (45, 70, 71). As a consequence, sub-pial lesions may be considered as typical of cortical TLO, thus defining a “*TLO-pathy*,” thereby adding a major conceptual brick to the understanding of CNS pathology. We acknowledge that these lesions have been reported only once, but one should remember how long it took to identify these key lesions in MS and that they were never explicitly tracked in other pathological settings. Acknowledging also that CNS pathologies involving intrathecal inflammation are either exceptional (like tropical infections) or too severe to be followed up for decades, MS is nowadays by far the most common cause of chronic intrathecal inflammation. We suspect that this epidemiological fact has biased observation by associating chronic intrathecal inflammation with MS, not because the association is specific but simply because it is statistically more likely. From a theoretical point of view, it could be important to longitudinally monitor non-MS patients with chronic intrathecal inflammation (implying TLO presence) in order to assess the possible development of progressive sub-pial lesions and brain atrophy reminiscent of the MS progressive phase. This would provide proof of TLO-pathy as a valuable new therapeutic target in all disorders involving intrathecal inflammation, including the still intractable progressive phase of MS.

## Can Non-Specific Intrathecal Synthesis Shed Light on Time of MS Onset?

### Influence of immunization schedule

Uncertainty remains about the timing of MS onset in the chronology of infantile MRZ infections or vaccination in the case of rubella, measles, and mumps. Moreover, most studies are dedicated to potentially neuro-infectious agents (measles, rubella, VZV, HSV, EBV, chlamydia), which obscure the analysis since one cannot exclude past subtle CNS infections. What would the results be for common infectious agents devoid of neurotropic effect (i.e., human papillomavirus, anti-tetanus toxin vaccine)? One might regret that anti-tetanus toxin, which is a marker of non-specific lymphoid IgG secretion commonly used in immunology, has never been tested in CSF. Is the intrathecal synthesis influenced by the schedule of immunizations (by infectious agents or vaccine) related to the time of MS onset? In other words, is the chance of acquiring non-specific intrathecal synthesis modified by the time of immunization, whether it occurs before, around, or after the clinical onset of MS? Once activated, B-cells have matured to PC in secondary lymphoid organs in response to an antigenic challenge, and long-lived PC migrate to bone marrow where they definitely home in nests providing a metabolic support. In this way, CNS may only recruit some PC during their short time frame of blood circulation succeeding B-cell differentiation. As a consequence, it may be that more than being an immune scar from early infection or immunization (6), non-specific intrathecal synthesis recapitulates and freezes the major antigenic challenges endured by the patient since MS onset. Elevated AI should be more common in protracted chronic CNS infections than in acute infection: an MRH reaction was described in a chronic case of neuro-borreliosis whereas no AI was elevated (except against borrelia) in an acute infection (58). There have been three case reports of MS with an initially normal AI that increased on a subsequent control lumbar puncture: two cases corresponded to current immunization against rubella and one case to a fresh VZV infection (72). Monitoring the differential intrathecal response to vaccines received early in life (i.e., anti-tetanus toxin) and those given later (i.e., hepatitis A/B, human papillomavirus) may help to demonstrate this hypothesis.

### Reassessing the paradox of low intrathecal anti-EBV reaction

Besides opening the debate on the significance of EBV infection in MS (73, 74), we now focus on intrathecal reaction against EBV (52). In a cohort of 24 CIS/MS patients selected for known intrathecal synthesis against EBV, *F*_s_ was in the same range against EBV (median 0.65%, range 0.01–4.78%) as against MRZ (52). One outlier patient had an *F*_s_ anti-EBV of 18.2%. When considering IgG against EBV, absolute levels are higher in most of MS patients than controls, both in serum and in CSF (75), although high CSF levels are more likely linked to blood-barrier dysfunction than to intrathecal synthesis (76). However, anti-EBV intrathecal values have shown that an unexpectedly small proportion of patients have intrathecal synthesis (52, 56, 77–79): for example, anti-VCA AI was elevated in 2/80 MS patients and anti-EBNA1 in 5/80, but there was no difference with control groups (77). In a study focusing on anti-EBNA1, intrathecal synthesis occurred in 5/76 MS and in 13/75 controls (78). In CIS patients (optic neuritis) sampled within 1 month after clinical onset, only 2/36 (6%) had an elevated AI (*F*_s_ 0.12–0.41%), whereas all were seropositive (52). In another study including 43 childhood and 50 adult onset MS, intrathecal synthesis occurred against measles, rubella, and VZV in 30–60% of pediatric and adult patients, whereas synthesis against anti-EBNA1 and anti-VCA occurred respectively only in 21 and 14% in the pediatric group and 8 and 2% in the adult group (80). Moreover, AI against EBV is sometimes twofold lower than AI against each MRZ component (80) but similar elsewhere (52). A difference in seroprevalence can be ruled out. Measles and rubella seroprevalence resulting either from natural infection or vaccination exceed 90% in most populations and vaccination campaigns started decades before (15), varicella seroprevalence exceeds 90% in all European populations and EBV seroprevalence is virtually complete in MS patients.

The lower than expected intrathecal response against EBV is not consistent with the strong correlation linking high serum anti-EBV levels and MS activity. In the light of this finding, such an extreme discrepancy can be interpreted as a strong clue for EBV infection preceding MS clinical onset (52). Similarly, one can suppose that EBV infection triggers a strong humoral response during which the homing of EBV-specific long-lived PC mostly precedes the onset of intrathecal inflammation and the formation of brain TLO. As a consequence, EBV-specific PC have less likelihood of being recruited by brain TLO, and therefore intrathecal AI against EBV remains paradoxically low. A different interpretation may involve a driving role of EBV-infected B-cells and PC (81), potentially modifying their ability to secrete antibodies against EBV antigens. The peculiar relation of EBV with MS pathology is reinforced by the demonstration of a high intrathecal EBV-specific CD8+ cytotoxic activity only early in MS patients, without recruitment of CD8+ cells against different targets (CMV-specific CD8+ cells) (82). Longitudinal studies of patients’ CSF for MRZ and EBV may help to assess whether the paradox of the low anti-EBV reaction is attributable to the schedule of primary infection or to a key pathophysiological point.

## Deriving Practical Consequences for MS Care. Can We Infer CNS TLO from a Simple CSF Analysis?

A growing set of evidence points to a central role of TLO in the formation and maintenance of intrathecal synthesis. Moreover, cortical TLO may play a key role in progressive MS (1, 42, 83, 84). Yet even though TLO structures may reach a considerable diameter (up to 1.1 mm), which may make them a target for future neuroimaging, most of them are barely detectable (about 50–100 cells) even after a thorough pathological examination (85). Unless uncovering a specific marker, TLO confirmation by conventional MRI seems a flimsy hope and biological techniques may be more advisable. Is there a CSF biological marker of TLO presence or persistence?

Tertiary lymphoid organs are able to support local clonal proliferation of B- and T-cells and provide survival niches for PC. T-cell clonal response has been demonstrated to be both “private” to brain regions and “public” since it is shared throughout the brain in all MS patients, and TCR clonotypes are strictly private to patients (46). Clonotype analysis of B-cell repertoire in multiple anatomical brain areas, including meningeal aggregates and CSF, showed a large overlap between local repertoires. As a consequence, analysis of the CSF clonotype repertoire gives a representative sample of the meningeal repertoire (86, 87). The CSF B-cell family germline repertoire is strongly biased to VH4 and VH3 (88) and private clonotypes are expanded from single ancestors (89, 90). Somatic hypermutations in CDR within RGYW/WRCY motifs of IgG (47), which are targeted by the activation-induced cytidinedeaminase (AICD) specifically expressed in lymphoid organs, suggest that most of these lineage cells have undergone a local germinal center reaction (91). All these indirect arguments strongly suggest the presence of TLO but necessitate high-level biological tests unavailable in routine practice. In fact, intrathecally produced IgG – elevated index, specific *F*_s_, OCB – is one of the major terminal products of TLO production and seems to be a valuable indirect hallmark of CNS TLO. To date, no therapy has reversed acquired intrathecal synthesis in MS. A series of 76 patients treated by natalizumab offers one exception: 16% were controlled negative for OCB after treatment, and the proportion of patients with an intrathecal synthesized fraction in the normal range increased from 20 to 45% (44). As a consequence, we propose that intrathecal IgG secretion could be used to monitor future TLO-targeted therapies.

We therefore hypothesize that intrathecal immune suppression should negate intrathecal IgG secretion by clearing CNS TLO (1). Clinical trials using intrathecal drugs offer a real hope to cure progressive MS. However, these trials were designed to use intrathecal rituximab. A pitfall of this treatment may be the resistance of CD20+ B-cells nested in TLO as observed in rheumatoid arthritis, and the persistence of local IgG secretion as observed in serum (1, 92). In fact, after intravenous + intrathecal rituximab treatment, a complete B-cell depletion is obtained in serum but depletion is still incomplete in CSF at 3 months: −85% for B-cells, −82% for T-cell (93). Results for IgG secretion and clinical/radiological data are not yet available. Although incomplete, these results suggest that intrathecal rituximab is a first step, but that it may not be sufficient alone to eradicate CNS TLO.

## Conclusion

Although typical of MS, the non-specific intrathecal reaction has long been a theoretical issue. We propose to consider this non-specific reaction as a common physiological property of intrathecal inflammation, involving the general properties of TLO located in the CNS. We extend this concept to (non-MS) CNS autoimmune and infectious disorders associated with intrathecal synthesis. Moreover, we propose that the cortical lesions associated with TLO observed in MS are not specific for MS but for TLO. We therefore propose the concept of “TLO-pathy” to describe cortical lesions associated with the presence of TLO. The concept of non-specific secretion deserves further study to clarify the influence of immunization schedule.

## Conflict of Interest Statement

The author declares that the research was conducted in the absence of any commercial or financial relationships that could be construed as a potential conflict of interest.
